# Accuracy of reported service use in a cohort of people who are chronically homeless and seriously mentally ill

**DOI:** 10.1186/s12888-016-0758-0

**Published:** 2016-02-25

**Authors:** Julian M. Somers, Akm Moniruzzaman, Lauren Currie, Stefanie N. Rezansoff, Angela Russolillo, Milad Parpouchi

**Affiliations:** Somers Research Group, Faculty of Health Sciences, Simon Fraser University, Burnaby, Canada

## Abstract

**Background:**

Self-reported service use is an integral feature of interventional research with people who are homeless and mentally ill. The objective of this study was to investigate the accuracy of self-reported involvement with major categories of publicly funded services (health, justice, social welfare) within this sub-population.

**Methods:**

Measures were administered pre-randomization in two randomized controlled trials, using timeline follow back with calendar aids for Health, Social, and Justice Service Use, compared to linked administrative data. Variables examined were: psychiatric admissions (both extended stays of more than 6 months and two or more stays within 5 years); emergency department visits, general hospitalization and jail in the past 6 months; and income assistance in the past 1 month. Participants (*n* = 433) met criteria for homelessness and a least one mental illness.

**Results:**

Prevalence adjusted and bias adjusted kappa (PABAK) values ranged between *moderate* and *almost perfect* for extended psychiatric hospital separations (PABAK: 0.77; 95 % confidence interval (CI) = 0.71, 0.83), multiple psychiatric hospitalizations (PABAK = 0.50, 95 % CI = 0.41, 0.59), emergency department visits (PABAK: 0.77; 95 % CI = 0.71, 0.83), jail (PABAK: 0.74; 95 % CI = 0.68, 0.81), and income assistance (PABAK: 0.82; 95 % CI = 0.76, 0.87). Significant differences in under versus over reporting were also found.

**Conclusions:**

People who are homeless and mentally ill reliably reported their overall use of health, justice, and income assistance services. Evidence of under-reporting and over-reporting of certain variables has implications for specific research questions.

ISRCTN registry: 57595077 (Vancouver at Home Study: Housing First plus Assertive Community Treatment versus congregate housing plus supports versus treatment as usual); and 66721740 (Vancouver at Home study: Housing First plus Intensive Case management versus treatment as usual).

## Background

Individuals who are both mentally ill and homeless are a major health policy focus internationally [1, 2]. Several multi-site randomized controlled trials have been mounted, with overlapping objectives that include diversion from health and justice services and reductions in the associated costs among samples defined by chronic homelessness and serious mental illness [3–5]. Self reported service involvement is commonly used as the basis for research ranging from national surveys [6] to the aforementioned experimental designs, and the resulting data are used to determine costs of care [7] as well as intervention outcomes.

Although some research has investigated the accuracy of self-reports among people who are either mentally ill [8] or homeless [9], few studies have examined the validity of self-reported service use in samples that are *both* homeless and seriously mentally ill. Moreover, previous research investigating validity of self-report in this population has used key informant responses as the comparator [10] rather than administrative sources. Research on shelter using veterans in the United States found only “moderate” concordance between self-reported and administrative data, leading the authors to recommend greater reliance on administrative records in research [11]. An earlier study among “skid row alcoholics” concluded that the unreliability of self-reports undermined the validity of research investigating employment, arrests, and treatment outcomes in this population [12]. More recently Clifasefi and colleagues [13] investigated the validity of self-reported public health utilization among chronically homeless individuals with severe alcohol problems and found fair-moderate agreement with administrative data for shorter (30 day) periods but inadequate agreement for longer (3 years) timeframes. Several recent studies with homeless and mentally ill adults examine self-reported events spanning up to 5 years [3, 4, 14].

The validity of self-reported information concerning service encounters may be compromised due to the severity of mental illness [15], or to common correlates of homelessness such as cognitive impairment [16] or substance use [17]. Moreover, questions concerning frequently used services may span several sectors including health, justice, and social assistance, with potential differences in social desirability or risks associated with disclosure [18]. For example, homeless and uninsured individuals are sometimes perceived as contributing to excessive emergency department visits [19], creating a negative bias. Jail and criminal charges may have an adverse impact on access to healthcare [20] as well as housing and support services. Furthermore, the validity of recall for events longer than 6 months previous may be particularly low [16]. Rosen and colleagues [15] compared self-reported receipt of social security income with administrative data for 7220 homeless people with mental disorders and found that 41 % of those who reported receiving benefits were unconfirmed by administrative sources. Moreover, the authors found that self-reported income assistance was more likely to be unverified among clients with psychotic disorders and longstanding substance use. These findings raise important questions regarding the use of self-reported service encounters for the purposes of calculating intervention outcomes as well as for identifying service priorities and gaps in care for members of a highly vulnerable population.

The use of administrative data as a gold standard measure of service use is often unwarranted, as these databases may be subject to inaccuracy and incompleteness [21, 22]. However, in settings with robust and centralized health and social welfare systems, administrative data systems reflect relatively complete records of services, and can be used to assess the validity of self-reports [22, 23].

### Aims of the study

The present study examined agreement between self-reported and administrative data for healthcare, corrections, and income assistance in a sample of homeless and severely mentally ill individuals. We hypothesized that agreement regarding hospital admissions would be lower for longer periods of recall than for shorter periods and that jail and emergency department visits would be under-reported due to low social desirability.

## Methods

### Participants

#### Ethics, consent and permissions

This study was reviewed and approved by the Research Ethics Board at Simon Fraser University. Participants were enrolled in the Vancouver at Home Study, which is comprised of two randomized controlled trials, ISRCTN registry: 57595077 (Vancouver at Home Study: Housing First plus Assertive Community Treatment versus congregate housing plus supports versus treatment as usual) and 66721740 (Vancouver at Home Study: Housing First plus Intensive Case management versus treatment as usual).

#### Consent to publish

Participants provided consent for the dissemination of results, and were asked to provide separate consent for investigators to receive administrative records from agencies responsible for health, justice, and social welfare services.

The present study exclusively examined data collected prior to randomization. Eligibility criteria included legal adult status (19 years of age or older), presence of a current mental disorder on the MINI International Neuropsychiatric Interview ([24]; MINI) and being absolutely homeless or precariously housed. Mental disorder status was confirmed through written diagnosis from physicians or other service providers wherever possible. We defined “absolutely homeless” as living on the streets or in a shelter for at least the past seven nights with little chance of obtaining secure accommodation, and “precariously housed” as living in a rooming house, hotel or other form of transitional housing with at least two episodes of absolute homelessness in the past year.

Recruitment of participants involved close collaboration with over 40 community-based agencies in Vancouver. Among those, the major sources of recruitment were: homeless shelters; drop-in centers; homeless outreach teams; hospitals; community mental health teams and criminal justice programs. Methodological details not included in the current study such as additional interviews and measures have been published separately [14].

Participants were invited to complete an eligibility screener as well as written informed consent prior to enrolment in the study. Our protocol for conducting informed consent was developed through pre-trial field-testing, including cognitive interviewing to ensure participant comprehension [25]. Interviews were discontinued if participants’ mental status appeared to be compromised by acute symptoms or substance use. Following the provision of consent, participants completed interviewer administered baseline questionnaires addressing: socio-demographic characteristics, symptoms of mental illness, substance use, and service use history. Interviewers were trained and supervised in the administration of all scales, including the use of calendar prompts in association with items requesting recall for events over different periods of time. Cash honoraria were provided for the screening questionnaire (**$**5.00) and the baseline interview (**$**25.00).

### Measures

#### Administrative data

We examined linked administrative data spanning three provincial government ministries responsible for: health services; justice; and income assistance. Residents of British Columbia are required to enroll with the Provincial Medical Services Plan. Hospital admissions and physician services are reported to the Ministry of Health, along with diagnostic details related to each admission or outpatient visit. The Ministry of Social Development and Social Innovation administers and records financial support to citizens based on demonstration of need, including disability and shelter payments. Details of correctional services (e.g., jail) are maintained by the Ministry of Justice. Mental and behavioural disorders from the International Classification of Diseases (ICD-10) were examined for physician diagnosed mental disorders (excluding psychoactive substance use) associated with hospitalization (F00-09; F20-99).

#### Availability of data and materials

Use of these linked data is governed by Information Sharing Agreements between the partnering ministries and the host university. Access to data is subject to police security clearance, restricted to a designated secure off-line environment and other provisions to protect privacy. Additional details concerning these variables are available from the corresponding author, and have been presented elsewhere [14, 26].

#### Self-reported data

The present analyses include the following socio-demographic variables, which were collected at baseline: gender; age; ethnicity (Aboriginal, White, Other); education; lifetime duration of homelessness; age first homeless; mental disorder status (type, severity, number of diagnoses); substance use disorder and daily substance use. We defined “severe” mental disorders on the basis of current (i.e. past month) psychosis, mood disorder with psychotic features, and hypomanic or manic episode, identified through the MINI. Substance dependence was also identified using the MINI. Timeline follow-back and calendar prompts were used to elicit details of service use associated with health, justice, and social welfare prior to recruitment.

### Statistical analysis

Descriptive statistics (mean and standard deviation for continuous variables; frequency and percentages for categorical variables) were used to characterize the study population. We used independent sample t tests to compare numerical variables (such as age at recruitment and homeless duration) and Pearson’s chi square test to compare categorical data (such as gender and ethnicity) between groups.

As a measure of agreement between two sources of records (self-reported vs. administrative data), we reported the simple percent agreement, Cohen’s kappa coefficient, and prevalence-adjusted bias-adjusted kappa (PABAK). We first calculated Cohen’s kappa, a widely used statistic of agreement for categorical data in clinical research. Although kappa is a more robust measure than simple percent agreement, the magnitude of kappa can be influenced by several factors including the prevalence of the condition of interest and bias, which refers to the extent of disagreement on the proportion of positive cases. Kappa will be underestimated if the prevalence index is high (i.e, prevalence is either very high or very low) and will be overestimated if the bias index is high [27, 28]. Therefore, we also calculated PABAK to better account for the influence of prevalence and bias. Several recent studies reported PABAK as a measure of agreement [29–32]. We reported 95 % Confidence Intervals for both Cohen’s kappa and PABAK.

We used Landis and Koch’s [33] classification to evaluate the strength of agreement, which is as follows: slight (0–0.20), fair (0.21–0.40), moderate (0.41–0.60), substantial (0.61–0.80), and almost perfect agreement (0.81–1.0). Because we assessed the agreement between different types of information, we hypothesized a social desirability bias for participants’ self-report. To investigate this phenomenon, we conducted McNemar tests of marginal homogeneity to identify any significant systematic difference in terms of disagreement (under-reporting vs. over-reporting) between self-report and administrative records. All reported *p* values were two sided. IBM SPSS Statistics 22.0 [34] and Stata 13 [35] were used to conduct these analyses.

## Results

The characteristics of participants who consented to accessing administrative data and whose records could be matched (*n* = 433) were compared to the full sample (*n* = 497) on a number of socio-demographic characteristics (see Table 1). The overall pattern of findings indicates that the eligible sample is broadly representative of the larger cohort. Members of the eligible sample were roughly 41 years of age and were first homeless about 11 years earlier. Nearly three quarters (74 %) were male, and participants self-identified as White (54 %), Indigenous (16 %), or Other ethnicity (30 %), comprised of Asian, African, Caribbean, Latin American, Middle Eastern, Mixed, or Other categories. The majority of participants met criteria for bipolar disorder, schizophrenia or both (“Severe cluster”; 72 %) as well as Substance Dependence (58 %). Table 1 also shows the percentage of the sample that self-reported: having been in hospital for more than 6 months (10 %) and more than two times (50 %) in the past 5 years; having been hospitalized (43 %), in jail (14 %), or charged with an offence (24 %) in the last 6 months; and having received either disability or income assistance (94 %) in the past month.

**Table 1:** Socio-demographic characteristics of ‘At home’ participants by consent status at enrolment visit

Variable	The entire sample (*n* = 497) *n* (%)/mean (SD)	Eligible sample^a^ (*n* = 433) *n* (%)/mean (SD)	Not eligible sample^b^ (*n* = 64) *n* (%)/mean (SD)	*P* value^c^
Age at randomization (in years)	40.8 (11.0)	40.8 (11.0)	41.4 (11.0)	0.682
Age of first homelessness (in years)	30.3 (13.3)	30.1 (13.4)	31.9 (12.6)	0.301
Female gender	134 (27)	112 (26)	22 (34)	0.165
Ethnicity				
Aboriginals	77 (16)	70 (16)	7 (11)	0.054
White	280 (56)	235 (54)	45 (70)	
Other	140 (28)	128 (30)	12 (19)	
Incomplete High School	280 (57)	247 (57)	33 (52)	0.376
Single/Never Married	343 (70)	293 (68)	50 (79)	0.071
Need level (high)	297 (60)	255 (59)	42 (66)	0.305
Housing first interventions	297 (60)	257 (59)	40 (63)	0.632
Lifetime duration of homelessness (in months)	60.2 (70.3)	58.3 (64.8)	72.9 (99.8)	0.124
Longest episode of homelessness (in months)	30.9 (40.1)	30.4 (39.5)	34.1 (44.4)	0.498
Less severe cluster of mental disorders	264 (53)	235 (54)	29 (45)	0.180
Severe cluster of mental disorders	363 (73)	311 (72)	52 (81)	0.113
Suicidality (high)	87 (17)	79 (18)	8 (12)	0.259
Substance dependence	288 (58)	252 (58)	36 (56)	0.768
Daily substance use	143 (29)	131 (30)	12 (19)	0.064
Mental health severity/CSI score (per unit)	37.2 (12.5)	37.4 (12.5)	35.8 (12.9)	0.371
Chronic Medical Conditions (3 or more)	344 (69)	305 (70)	39 (61)	0.124
Blood-borne Infectious Disease (HIV, Hep. B or C)	157 (32)	139 (32)	18 (29)	0.603
Hospitalized for mental illness in last 5 years^d^				
Over 6 months	57 (11)	44 (10)	13 (20)	**0.017**
Two times or more	253 (51)	215 (50)	38 (59)	0.146
Hospitalization in last 6 months	212 (43)	187 (43)	25 (39)	0.533
Any formal charge in last 6 months	111 (23)	101 (24)	10 (16)	0.172
Jail in last 6 months	69 (14)	62 (14)	7 (11)	0.465
Source of income (disability or income assistance) in past month	469 (94)	408 (94)	61 (95)	0.725

^a^Out of 497 participants, 433 provided consent to access to administrative health data and were linkable to health records

^b^Out of 64 participants, 60 didn’t consent to access to administrative health data and 4 provided consent, but were unlink-able to health records

^c^ *P* values based on comparisons of characteristics between eligible participants and non-eligible participants in the entire sample

^d^Didn’t know was considered as no

Bold signifies statistical significance

Table 2 presents the simple percent agreement and agreement statistics (Cohen’s kappa coefficient and PABAK) between the self-reports and administrative records across specific domains of public service utilization. Based on Cohen’s kappa coefficient, five of the six variables examined showed a moderate agreement (jail: kappa = 0.55, 95 % confidence interval (CI) = 0.46, 0.64; multiple psychiatric hospitalizations: kappa = 0.50, 95 % CI = 0.41, 0.59; disability or income assistance: kappa = 0.47, 95 % CI = 0.38, 0.56; ER visit: kappa = 0.44, 95 % CI = 0.34, 0.53; any hospitalization: kappa = 0.44, 95 % CI = 0.34, 0.53) and the remaining variable (6 months psychiatric hospitalization) indicated a poor agreement (kappa = 0.21, 95 % CI = 0.12, 0.30). As expected, some of these variables demonstrated a high prevalence index (6 months psychiatric hospitalization: 0.84, disability or income assistance: 0.81 and jail: 0.66) associated with the fact that these variables represented either rare (6 months psychiatric hospitalization) or very common conditions (disability or income assistance) in the sample. Bias index was minimal across all variables except for multiple psychiatric hospitalizations. When adjusted for imbalance caused by prevalence differences and bias, kappa values (PABAK) increased substantially for variables addressing 6 month psychiatric hospital separations (PABAK: 0.77; 95 % CI = 0.71, 0.83), disability or income assistance (PABAK: 0.82; 95 % CI = 0.76, 0.87) and jail (PABAK: 0.74; 95 % CI = 0.68, 0.81). Based on PABAK, these variablesshowed substantial (jail and 6 months psychiatric hospitalization) or almost perfect (disability or income assistance) agreement. For other variables, PABAK values showed the same moderate level of agreement as measured by Cohen’s kappa coefficient.

**Table 2:** Agreement between self-report and administrative records of public service utilization among ‘At Home’ study participants (*n* = 433^a^)

Variables	Recall period	Agreement			Disagreement			Observed kappa (95 % CI)	Prevalence index, Bias index	PABAK^b^ (95 % CI)	McNemar test, *p* value
		Overall	Both yes	Both no	Overall	Over reporting	Under reporting				
Hospitalized over six months for mental illness	5 years	88 %	2 %	86 %	12 %	8 %	4 %	0.21 (0.12, 0.30)	0.84, 0.05	0.77 (0.71, 0.83)	**0.007**
Hospitalized at least two times for mental illness	5 years	75 %	30 %	45 %	25 %	19 %	6 %	0.50 (0.41, 0.59)	0.14, 0.13	0.50 (0.42, 0.58)	**<0.001**
Any Hospitalization	6 months	73 %	28 %	45 %	27 %	16 %	11 %	0.44 (0.34, 0.53)	0.18, 0.04	0.46 (0.37, 0.54)	0.117
Any emergency room visit^c^	6 months	73 %	46 %	27 %	27 %	9 %	18 %	0.44 (0.34, 0.53)	0.19, 0.08	0.45 (0.36, 0.54)	**0.002**
Jail^d^	6 months	87 %	11 %	76 %	13 %	4 %	9 %	0.55 (0.46, 0.64)	0.66, 0.06	0.74 (0.68, 0.81)	**0.001**
Income source: disability or income assistance	Past month	91 %	86 %	5 %	9 %	8 %	1 %	0.47 (0.38, 0.56)	0.81, 0.07	0.82 (0.76, 0.87)	**<0.001**

^a^Out of 497 participants, 433 provided consent to access to administrative data and were linkable to health records

^b^PABAK means prevalence adjusted and bias adjusted kappa

^c^Restricted to 387 participants

^d^Analysis was restricted to 428 participants since consent status varied across public service domains

Table 2 also presents findings from the McNemar test, which was conducted to identify any systematic difference in terms of disagreement (under-reporting vs. over-reporting). Five of the six variables examined resulted in significant disagreement between sources. Participants significantly over-reported having been hospitalized for 6 months or more for a mental illness (8 % vs. 4 %, *p* = 0.007) and having been hospitalized at least two times for a mental illness (19 % vs. 6 %, *p* < 0.001) – both in the previous 5 years. Participants significantly under-reported having been to an emergency department (18 % vs. 9 % *p* = 0.002) or to jail (4 % vs. 9 %, *p* = 0.001) within the past 6 months. Finally, participants significantly over-reported having received disability or income assistance in the past month (8 % vs. 1 %, *p* < 0.001). There was no significant disagreement between self-report and administrative data with respect to having been hospitalized for any reason in the past 6 months.

## Discussion

Our findings reveal *moderate* to *almost perfect* [33] agreement between self-reported service use and corresponding information from administrative sources. To the best of our knowledge, this is the first study to directly compare self-reported and administrative data for multiple domains of public service in a single large sample meeting criteria for chronic homelessness and serious mental illness. These results support the validity of self-report data as the basis for research examining changes in service use and related costs with this sub-population. In so doing they support the role of people who experience both mental illness and homelessness as participants in the production of knowledge.

Our results hold particular relevance for experimental trials such as those recently implemented in North America [3] and Europe [4], which rely on self-reports to investigate the impact of interventions on service use among people who are both homeless and mentally ill. More specifically, the aforementioned trials incorporated items concerning self-reported psychiatric hospitalization in the past 5 years as inclusion criteria - items that our results specifically corroborate.

Previous research among homeless people with chronic alcohol problems employed a similar design to the current study and reported “inadequate” recall over a 3-year period [13]. These findings and those of earlier research among alcoholics [12] may be attributable to the cognitive effects of chronic alcohol exposure [36]. By contrast, our results suggest that individuals who have experienced longstanding homelessness (i.e., 10 years) alongside psychosis or bipolar disorder are nevertheless reliable reporters concerning events in both recent (past month) and distant (5 years) history.

Despite the overall level of agreement between data sources, we identified significant systematic differences in under-reporting versus over-reporting on five out of six variables. Questions related to psychiatric hospitalization during the past 5 years (whether a single long admission or multiple admissions) were associated with significant over-reporting. In contrast, questions related to emergency department visits or jail admissions during the past 6 months were associated with significant under-reporting. Finally, having received income assistance in the past month was significantly over-reported, a result that is consistent with previous research on this sub-population [15]. We cannot specify the reasons for disagreement between sources, or why some variables were over-reported while others were under-reported. Nevertheless, the over-reporting of psychiatric hospitalizations in the preceding 5 years may have been due to the difficulty of recalling events over a lengthy period, and may have elicited positive responses corresponding to highly salient events that occurred longer than 5 years in the past. The under-reporting of jail in the preceding 6 months may reflect social desirability as well as stigma associated with incarceration. Homeless individuals may use emergency departments for primary healthcare, and may therefor under-report visits that do not involve emergency complaints. These findings are particularly relevant to research that focuses on specific services (e.g., jail) where systematic over or under-reporting could bias results. They are also relevant to clinical settings where biased responding (e.g., over-reporting of income assistance) may lead to oversights in care planning for patients.

Strengths of our study include: the use of verified administrative data; multiple categories of service; administrative records spanning a long-duration (i.e., up to 5 years); pre-testing of interview questions [25]; and use of calendar aids to strengthen recall. Limitations of our study include: unaccounted for errors associated with administrative records (e.g., coding errors); potential use of aliases when receiving care; and unrecorded events due to not having identification.

## Conclusions

Our study found that individuals who experience chronic homelessness and serious mental illness are accurate historians regarding their encounters with public services. We observed high levels of agreement between administrative records and self-report for healthcare, jail, and welfare support spanning periods of time from 1 month to 5 years. Significant over-reporting (e.g., psychiatric hospitalization) and under-reporting (e.g., jail) was specific to individual service areas and warrants caution in studies that focus on these particular domains.
